# Associations between the built environment and physical activity among adults with low socio-economic status in Canada: a systematic review

**DOI:** 10.17269/s41997-020-00364-9

**Published:** 2020-08-24

**Authors:** Chelsea D. Christie, Anna Consoli, Paul E. Ronksley, Jennifer E. Vena, Christine M. Friedenreich, Gavin R. McCormack

**Affiliations:** 1 Department of Community Health Sciences, Cumming School of Medicine, University of Calgary, TRW 3rd floor, 3280 Hospital Drive NW, Calgary, Alberta T2N 4Z6 Canada; 2 CancerControl Alberta, Alberta Health Services, Richmond Road Diagnostic and Treatment Centre, 1820 Richmond Road SW, Calgary, Alberta T2T 5C7 Canada; 3 Department of Cancer Epidemiology and Prevention Research, CancerControl Alberta, Alberta Health Services, 2210 2nd St SW, Calgary, Alberta T2S 3C3 Canada; 4 School of Architecture, Planning and Landscape, University of Calgary, 2500 University Dr NW, Calgary, Alberta T2N 4N1 Canada

**Keywords:** Physical activity, Built environment, Socio-economic status, Neighbourhood, Activité physique, environnement bâti, statut socioéconomique, quartier

## Abstract

**Objective:**

To synthesize literature on the associations between the built environment and physical activity among adults with low socio-economic status (SES) in Canada.

**Methods:**

Using a pre-specified study protocol (PROSPERO ID: CRD42019117894), we searched seven databases from inception to November 2018, for peer-reviewed quantitative studies that (1) included adults with low SES living in Canada and (2) estimated the association between self-reported or objectively measured built characteristics and self-reported or objectively measured physical activity. Study quality was assessed using the Quality Assessment Tool for Observational Cohort and Cross-Sectional Studies. Findings were synthesized using a narrative approach.

**Synthesis:**

Of the 8338 citations identified by our search, seven studies met the inclusion criteria. Most studies included adults living in one province (Alberta, British Columbia, Ontario, or Quebec), with one study including a national sample. All studies were cross-sectional, and none controlled for residential self-selection. Sampling designs and data collection strategies were heterogeneous. Sample sizes ranged between 78 and 37,241 participants. Most studies measured SES using household income. Street connectivity, greenness, destination density, and walkability were positively associated with physical activity. Relative to the objectively measured built environment, associations between the self-reported built environment and physical activity were less consistent. Studies were of fair to good quality.

**Conclusion:**

Findings suggest that the neighbourhood built environment is associated with physical activity among adults with low SES in Canada. More rigorous study designs are needed to determine whether or not the built environment and physical activity are causally related within this vulnerable population.

## Résumé

**Objectif:**

Faire une synthèse de la littérature scientifique sur les associations entre l’environnement bâti et l’activité physique chez les adultes de faible statut socioéconomique (SSE) au Canada.

**Méthode:**

À l’aide d’un protocole d’étude préétabli (numéro d’identification PROSPERO : CRD42019117894), nous avons interrogé sept bases de données, entre l’inception de chacune et novembre 2018, pour repérer les études quantitatives évaluées par les pairs qui : 1) incluaient des adultes de faible SSE vivant au Canada; et 2) estimaient l’association entre les caractéristiques autodéclarées ou objectivement mesurées de l’environnement bâti et l’activité physique autodéclarée ou objectivement mesurée. La qualité des études a été évaluée à l’aide de l’outil Quality Assessment Tool for Observational Cohort and Cross-Sectional Studies. Nous avons fait la synthèse des constatations selon une démarche narrative.

**Synthèse:**

Sur les 8 338 citations repérées dans notre recherche, sept études répondaient aux critères d’inclusion. La plupart incluaient des adultes vivant dans une province (Alberta, Colombie-Britannique, Ontario ou Québec), et une étude comportait un échantillon national. Toutes les études étaient transversales, et aucune ne tenait compte de l’auto-sélection de la résidence. Les modes d’échantillonnage et les stratégies de collecte de données étaient hétérogènes. La taille des échantillons variait de 78 à 37 241 participants. La plupart des études mesuraient le SSE à l’aide du revenu des ménages. La connectivité des rues, la verdure, la densité des destinations et la marchabilité étaient associées positivement à l’activité physique. Par rapport à l’environnement bâti objectivement mesuré, les associations entre l’environnement bâti autodéclaré et l’activité physique étaient moins constantes. La qualité des études était de moyenne à bonne.

**Conclusion:**

Nos constatations indiquent que l’environnement bâti du quartier est associé à l’activité physique chez les adultes de faible SSE au Canada. Des protocoles d’étude plus rigoureux sont nécessaires pour déterminer s’il existe ou non une relation causale entre l’environnement bâti et l’activité physique dans cette population vulnérable.

## Introduction

Low levels of physical activity are associated with an increased risk of adverse health conditions, including cardiovascular diseases, hypertension, overweight and obesity, type II diabetes, depression, and some cancers (Lim et al. 2012; Fogelholm 2010; Warburton and Bredin 2017). Despite the risks associated with low physical activity, fewer than one in five adults meet the Canadian physical activity guidelines related to aerobic physical activity (≥ 150 min of moderate- to vigorous-intensity aerobic physical activity per week, in bouts of 10 min or more; Colley et al. 2018; Tremblay et al. 2011).

Findings from systematic reviews suggest that neighbourhood built characteristics, including sidewalks, pedestrian connectivity, land-use mix, residential density, and walkability (the combination of several built characteristics into a single index), are positively associated with physical activity (Hajna et al. 2015; Kärmeniemi et al. 2018; Ding et al. 2018; Ding and Gebel 2012; Barnett et al. 2017; Van Cauwenberg et al. 2011). While this evidence is promising, few reviews have synthesized studies examining relationships between the built environment and physical activity among specific subpopulations (Barnett et al. 2017; Van Cauwenberg et al. 2011). Furthermore, few reviews restrict to studies estimating built environment and physical activity associations in a single country (e.g., Schulz et al. 2018; Farkas et al. 2019). Findings from one country may not be generalizable to another due to socio-cultural, climatic, and geographical differences.

A paucity of studies have investigated whether or not the relationship between the built environment and physical activity differs according to socio-economic status (SES; Adkins et al. 2017). The environmental constraints and barriers affecting physical activity may differ according to SES. Developing a better understanding of determinants of physical activity for adults with low SES is crucial given they are at increased risk, relative to those with high SES, for several diseases associated with physical inactivity (Raphael 2016). Chudyk et al. (2015) proposed that people with low SES may benefit more from walkable environments because they may be more likely to rely upon free activities, such as outdoor walking. Alternatively, Adkins et al. (2017) suggested that, because they have limited transportation options, people with low SES might walk similar amounts regardless of whether they reside in low or high walkable environments. If people with low SES are differentially impacted by the built environment, this differential effect may partially explain why adults with low SES tend to report lower rates of physical activity compared with adults with higher SES (Pan et al. 2009).

Reviews synthesizing evidence on effect modification by SES on the built environment–physical activity relationship have drawn conflicting conclusions (Adkins et al. 2017; Smith et al. 2017; Pearce and Maddison 2011). One systematic review found that several built characteristics were positively associated with physical activity among US adults with low SES (Lovasi et al. 2009). The findings from this review, however, may not generalize to Canada, given that the distributions of poverty and neighbourhood influences on health differ between the two countries (Oreopoulos 2008). Furthermore, there are differences between Canada and the United States on factors that may impact active transportation decisions (walking and cycling), such as gasoline prices, levels of land-use mix within inner cities, median work trip distances, and cars per capita (Pucher and Buehler 2006). Finally, given that Canadian evidence might better inform local urban planning and public health-related policy and practice, the purpose of this systematic review was to synthesize evidence on associations between the built environment and physical activity among adults with low SES in Canada.

## Methods

We conducted this systematic review in accordance with the PRISMA (Preferred Reporting Items for Systematic Reviews and Meta-Analyses) guidelines (Moher et al. 2009) and followed a pre-specified study protocol (PROSPERO ID: CRD42019117894).

### Data sources and search strategy

We searched seven electronic databases (Medline, PsycINFO, Web of Science, SPORT Discus, Transport Research International Documentation (TRID), Urban Studies Abstracts, and Environment Complete) from inception of each database (i.e., 1888 to 1967) to November 1, 2018, with no language restrictions. In addition, we screened reference lists from previous reviews and other relevant publications from the authors’ libraries.

A systematic approach was used to create search queries for each database. Each search query included a series of keywords and subject term headings for three main components (Canada, built characteristics, and physical activity; see Appendix 1). The fourth component, SES, was considered at the full-text review stage as an inclusion criterion. The search strategies were verified for their completeness by an expert librarian. Only peer-reviewed journal articles were eligible. Two authors of this review (CC, AC) also scanned the reference lists of all articles selected in the primary search to identify any additional studies.

### Selection process

Initial title and abstract screening of the identified articles was performed independently by two reviewers (CC, AC) to determine eligibility for full-text review. This initial screen was intentionally broad and included any study reporting on the association between the built environment and physical activity among adults in Canada. We chose to wait until full-text screening to identify populations with low SES to avoid excluding studies that included individuals of various SES levels but tested for modification or stratified the results by SES (in which case the article would be eligible). Full-text screening was performed by two reviewers (CC, AC) to determine inclusion of the studies in the review.

### Eligibility criteria

A study was eligible for inclusion if it included (1) a sample or subsample of adults (≥ 18 years) with low SES living in Canada (urban or rural locations); (2) any self-reported or objectively measured neighbourhood built characteristic potentially supportive of physical activity; (3) any self-reported or objectively measured physical activity outcome; and (4) estimates of an association between the built environment and physical activity. Any author-identified indicators of low SES (e.g., low household or individual income, low education, or neighbourhood-level measures of disadvantage) were eligible. Inter-rater agreement was measured at the full-text screening stage and disagreements were resolved by consultation with another author (GM).

### Data extraction and quality assessment

Data extraction and quality assessment were completed independently by two reviewers (CC, AC). Data extracted included study design, sampling design, demographic information, and measures of SES, the built environment, and physical activity. Where available, effect measure estimates quantifying the relationship of interest were reported. When articles reported information for a subgroup that met the inclusion criteria (e.g., stratifying the results for high- and low-income individuals), only the information from the low-SES subgroup was extracted.

Study quality was assessed using a modified (8-item) version of the National Heart, Lung and Blood Institute (NHLBI) of Health Quality Assessment Tool for Observational Cohort and Cross-Sectional Studies (NHLBI 2014). Items that were relevant only for cohort studies were removed (e.g., related to loss to follow-up). Adjustment for confounding was considered sufficient if studies adjusted for at least one socio-demographic variable (e.g., age) and at least one behavioural variable (e.g., vehicle ownership). CC and AC independently used the tool to evaluate the quality of each study. Response options for the items were: “yes,” “no,” “not applicable,” “cannot determine,” or “not reported.” Individual items were used to assess risk of bias, which guided the overall rating for the quality of each study as “good,” “fair,” or “poor” (See Appendix 2).

### Synthesis of results

We grouped study results based on whether or not the built environment was objectively measured or self-reported. Study-specific results, including point estimates and confidence intervals, were reported in tabular format. To reduce potential bias due to confounding, only the most adjusted effect estimates were reported (when available). Heterogeneity of measures of the built environment and physical activity between studies meant that a meta-analysis was not possible. Therefore, we synthesized the study results using a narrative approach.

## Results

### Identification of studies

**Fig. 1:**
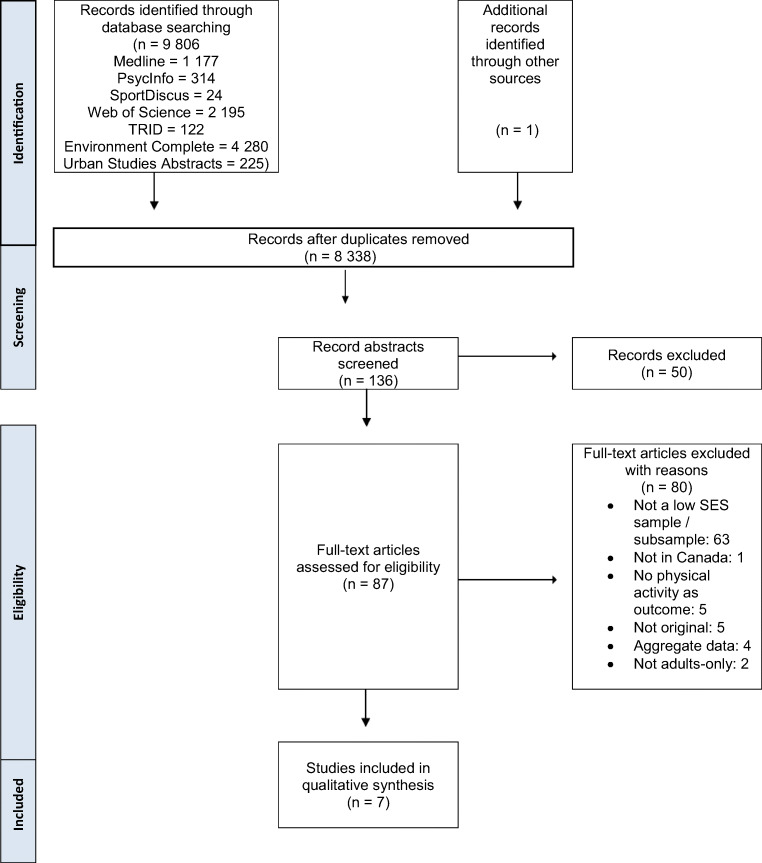
PRISMA flow diagram of screening process

The initial search yielded 9807 records. After duplicates were removed, 8338 records were screened at the title/abstract phase and 87 articles underwent full-text review. Inter-rater agreement (κ = 0.86) at the full-text screening stage was excellent (Altman 1990). Upon completion of the full-text screen, seven studies satisfied the inclusion criteria and were included in the review (Fig. 1).

### Summary of study characteristics

#### Sample and study design

All seven studies employed a cross-sectional design and were published between 2011 and 2017, with most (*n* = 5) published in 2015 or later (Table 1). Six articles (5 unique studies) had samples from a single province (Alberta, British Columbia, Ontario, or Quebec), and one study included national data (McMorris et al. 2015). All studies had samples from urban areas. One study used a simple random sampling strategy (McCormack et al. 2014), two articles (one unique study) used a stratified random sampling technique (Chudyk et al. 2017; Chudyk et al. 2015), and one study used both stratified random sampling and snowball sampling (Perez et al. 2011). Three articles (two unique studies) reported low SES adults participation rates of 8% and 38% (Perez et al. 2011; Chudyk et al. 2017; Chudyk et al. 2015). Participation rates for the low SES participants in four studies were not reported; however, whole sample participation rates of 16% (McCormack et al. 2014), 74% (Winters et al. 2015), and 80% (McMorris et al. 2015) were available. For the studies that reported a mean age for the low-SES participants (*n* = 3), the lowest was 39.3 years (Perez et al. 2011) and the highest was 74 years (Chudyk et al. 2015; Chudyk et al. 2017). Three studies included older adults only (≥ 65 years old; Chudyk et al. 2017; Chudyk et al. 2015; Winters et al. 2015) and one study included women only (Perez et al. 2011).

**Table 1:** Summary of study characteristics and sample socio-demographics for the 7 included studies

Study	Design	Province	Sample size	Age range (years)	Female (%)	Built environment (*data source*)	Physical activity (*data source*)	SES indicator
Chudyk et al. 2015	Cross-sectional	BC	150	65+	66	Walkability (*Street Smart WalkScore®*)	Transportation walking (*travel diary*)	Recipient of the Shelter Aid for Elderly Renters (SAFER)
Chudyk et al. 2017	Cross-sectional	BC	161	65+	63	Walkability (*Street Smart WalkScore®*) Self-reported walkability (*NEWS-A*)	Physical activity (*accelerometer*) Walking for transportation (*CHAMPS*)	Recipient of the Shelter Aid for Elderly Renters (SAFER)
McCormack et al. 2014	Cross-sectional	AB	762 low income 524 low education	NR	NR	Neighbourhood walkability type High, medium, low (*cluster analysis*)	Total neighbourhood-based PA (*interview and questionnaire*)	< $80,000 annual household income High school or less
McMorris et al. 2015	Cross-sectional	All	8963	NR	NR	Measures of greenness (*NDVI*)	Leisure physical activity (*CCHS*)	Two lowest income adequacy groups
Perez et al. 2011	Cross-sectional	ON	78 Group 1^a^ 110 Group 2^b^ 98 Group 3^c^ 91 Group 4^d^	18–89	100	Self-reported walkability (*NEWS-A*)	Sedentariness and total PA (*IPAQ*)	Reside in a specific multi-ethnic community with a high proportion of recent immigrants, low-SES residents, and crime; sample subdivided into 4 groups by vehicle access and being a native English speaker
Steinmetz-Wood and Kestens 2015	Cross-sectional	QC	37,241	NR	NR	Density of business and service destinations (*DMTI 2008*) Connectivity (*DMTI 2010*) Land-use mix (*DMTI 2007*)	Active transportation (*Montreal Origin-destination computer-assisted phone interview survey*)	< Median for Pampalon index of material disadvantage (neighbourhood level)
Winters et al. 2015	Cross-sectional	BC	305	NR	NR	Walkability (*Street Smart WalkScore®*)	Meeting PA guidelines through walking outside (*CCHS-HA*)	≤ $30,000/year (based on the median family income for Canadian older adults)

*AB*, Alberta; *BC*, British Columbia; *All*, all provinces; *ON*, Ontario; *QC*, Quebec

*NR*, not reported

*PA*, physical activity

*NEWS-A*, *Neighbourhood Environment Walkability Scale-Abbreviated*

*CHAMPS*, *Community Healthy Activities Model Program for Seniors survey*

*NDVI*, *Normalized Difference Vegetation Index*

*CCHS*, *Canadian Community Health Survey*

*IPAQ*, *International Physical Activity Questionnaire*

*CCHS-HA*, *Canadian Community Health Survey-Healthy Aging Cycle*

^a^Group 1, no vehicle access and non-native English speaker

^b^Group 2, no vehicle access and native English speaker

^c^Group 3, vehicle access and non-native English speaker

^d^Group 4, vehicle access and native English speaker

#### Socio-economic status indicators

Indicators of SES were mainly measured at the individual level (*n* = 5), with two studies using neighbourhood-level indicators. Individual-level SES indicators were different across the included articles, except for the two articles based on the same study, where low-income adults were operationalized as adults aged ≥ 60 years who qualified for a housing subsidy (Chudyk et al. 2017; Chudyk et al. 2015). Other individual-level SES indicators included household income (as a raw value for all participants or adjusted for household size) or level of education achieved (as high school or less). Of the two studies that assessed SES at the neighbourhood level, one defined a low-SES neighbourhood as a neighbourhood scoring below the median of neighbourhoods in the study based on the Pampalon index (Steinmetz-Wood and Kestens 2015), and the other selected a multi-ethnic Toronto community with a high proportion of recent immigrants, low-SES residents, and crime (Perez et al. 2011).

#### Built environment measures

Five studies included objectively measured built environment variables, while one study included self-reported walkability only (Perez et al. 2011), and one study included both objective and self-reported measures (Chudyk et al. 2017). The Street Smart Walk Score® was used in three studies (Chudyk et al. 2017; Chudyk et al. 2015; Winters et al. 2015). Walk Score® is calculated from the number of intersections, average block length, and distance to nearest amenities. McMorris et al. (2015) used the Normalized Difference Vegetation Index (NDVI) to assess degree of vegetation. One study used a cluster analysis with objectively measured geographic information systems (GIS) environment data (walkshed area, density of businesses and services, density of bus stops, sidewalk length, mix of park types, mix of recreational facilities, population density, pathway and cycleway length, and proportion of area as green space) to identify homogenous types of neighbourhoods based on their walkability (high, medium, low walkability; McCormack et al. 2014). Finally, one study estimated connectivity, land-use mix, and density of businesses and services using GIS (Steinmetz-Wood and Kestens 2015). The two studies that measured self-reported walkability used the abbreviated Neighbourhood Environment Walkability Scale (NEWS-A; Perez et al. 2011; Chudyk et al. 2017).

Operational definitions for neighbourhood size (i.e., the area around a participant’s home that was used to estimate the neighbourhood built environment) ranged from a 400-m buffer around the shortest path of a trip (Steinmetz-Wood and Kestens 2015) to a 1.5 mile distance from a participant’s home (Chudyk et al. 2017; Chudyk et al. 2015; Winters et al. 2015). The definition of neighbourhood also varied based on whether researchers used circular buffers (McMorris et al. 2015) or network buffers (McCormack et al. 2014) or used a participant determined neighbourhood (Perez et al. 2011). No studies considered length of neighbourhood residency.

#### Physical activity measures

Physical activity was most frequently measured through self-reports (*n* = 6), collected through questionnaires (*n* = 6) or telephone interviews (*n* = 1). Physical activity was also objectively measured via accelerometers (*n* = 1), and participation in or duration of various types of physical activity was assessed (Table 1). Only two studies used context or neighbourhood-specific measures of physical activity (Steinmetz-Wood and Kestens 2015; McCormack et al. 2014). Transportation-related physical activity was the most commonly measured type of physical activity.

#### Covariates

Most studies adjusted for age (*n* = 7) and sex (*n* = 6). Education, access to a vehicle/possession of a driver’s license, mobility, and dog ownership were also common covariates (*n* = 3). No studies adjusted for residential self-selection (i.e., participants choosing a neighbourhood matched to their physical activity preferences). Most studies collected data across multiple seasons; however, only one study statistically adjusted for seasonal variation in physical activity (McMorris et al. 2015). One study restricted data collection to a single season (Chudyk et al. 2017; Chudyk et al. 2015).

### Associations between the built environment and physical activity in low SES adults

Overall, the studies in this review reported 27 null associations (61%) and 17 significant associations (39%), all in the expected direction (i.e., consistent with previous evidence). Significant associations between the built environment and physical activity were found most consistently when the built environment was objectively measured (16/28 or 57%) vs self-reported (1/16 or 6%).

#### Objectively measured built environment

Sixteen positive associations and twelve non-statistically significant associations were found between the objectively measured built environment and physical activity within five unique studies (Table 2).

**Table 2:** Summary of the associations between the *objectively measured* built characteristics and physical activity in adults with low SES

Study	Built characteristics	Type of PA	Measurements of PA	Estimates (95% CI)
Chudyk et al. (2015)	*Street Smart Walk Score*®	*Walking for Transportation*	Average number of trips per day	IRR = 1.2 (1.12–1.29)*
Chudyk et al. (2017)	*Street Smart Walk Score*®	*Physical Activity*	*Physical Activity*	
		Total activity counts (TAC)	TAC number/day	*β* = 1.00 (0.96–1.04)
		Steps	Number of steps/day	*β* = −23 (−207–160)
		Light PA	Light PA minutes/day	*β* = −5.22 (−10.83–0.39)
		Moderate or Vigorous PA	MVPA minutes/day	*β* = 1.00 (0.92–1.09)
		*Walking for Transportation (TW)*	*Transportation Walking*	
		Participation	Participation (Yes/No)	OR = 1.45 (1.18–1.78)*
		Frequency	Number of TW/week	IRR = 1.03 (0.98–1.08)
		Duration	TW hours/week	*β* = −0.01 (−0.27–0.25)
McCormack et al. (2014)	*Neighbourhood*	*Total Neighbourhood Physical activity*	MET-minutes/week	
	*Walkability*	Low-Income Walkability HW vs LW		% difference = ~41%*
	High (HW)	Low-Income Walkability HW vs MW		% difference = ~42%*
	Medium (MW)	Low-Education Walkability HW vs LW		% difference = ~49%*
	Low (LW)	Low-Education Walkability HW vs MW		% difference = ~53%*
McMorris et al. (2015)	*NDVI Greenness*	*Leisure Physical Activity*	*Participation (Yes/No)*	
	Q1 (least green)			
	Q2		Q2 vs Q1	OR = 0.98 (0.85–1.13)
	Q3		Q3 vs Q1	OR = 1.17 (1.00–1.36)*
	Q4 (most green)		Q4 vs Q1	OR = 1.12 (0.95–1.33)
Steinmetz-Wood and Kestens (2015)	*Connectivity*	*Active Transportation*	*Participation (Yes/No)*	
	Q1 – Q4		Connectivity	
	*Land-use mix*		Q2 vs Q1	NR
	Q1 – Q4		Q3 vs Q1	OR = 1.12 (1.01–1.23)*
	*Density of destinations*		Q4 vs Q1	OR = 1.59 (1.43–1.76)*
	Q1-Q4		*Land-use mix*	
			Q2 vs Q1	NR
			Q3 vs Q1	NR
			Q4 vs Q1	NR
			*Destination density*	
			Q2 vs Q1	OR = 1.17 (1.08–1.27)*
			Q3 vs Q1	OR = 1.47 (1.35–1.61)*
			Q4 vs Q1	OR = 2.67 (2.41–2.95)*
Winters et al. (2015)	*Street Smart Walk Score*®	*Achieving PA guidelines through walking outside*	*Participation in sufficient PA (Yes/No)*	
	Continuous		Continuous	OR = 1.17 (1.07–1.27)*
	Categorical^a^		Categorical	
			2 vs 1	OR = 1.83 (1.16–2.88)*
			3 vs 1	OR = 1.95 (1.25–3.07)*
			4 vs 1	OR = 3.57 (1.62–7.87)*

*p < 0.05

*NR* not reported.

^a^1. Very car-dependent/car-dependent

2. Somewhat walkable

3. Very walkable

4. Walker’s paradise

~ indicates a value is approximate because it was estimated from a figure

Among older adults with low incomes, positive associations were found between Street Smart Walk Score® and frequency of daily transportation walking trips (Chudyk et al. 2015), participation in weekly transportation walking (Chudyk et al. 2017), and meeting physical activity guidelines (≥ 150 min per week) through outdoor walking (Winters et al. 2015). In contrast, Chudyk et al. (2017) found that Street Smart Walk Score® was not associated with total activity count (total volume of activity per day, across all intensities), number of steps per day, time spent in light or moderate-to-vigorous physical activity per day, nor frequency or duration of transportation walking per week.

For adults from low-income households, McMorris et al. (2015) found that level of neighbourhood greenness was positively associated with participation in any leisure time physical activity within the past three months. Among individuals from disadvantaged neighbourhoods, street connectivity and density of businesses and services were positively associated with higher odds of using active transportation for a weekday trip (Steinmetz-Wood and Kestens 2015). Finally, when neighbourhoods were categorized into types according to their objectively measured built characteristics, McCormack et al. (2014) found that weekly neighbourhood-based physical activity was higher for low-income individuals and low-education individuals residing in highly walkable vs medium- or low-walkable neighbourhoods.

#### Self-reported built environment

Two studies reported 16 associations between self-reported measures of the built environment and physical activity, and only one of these associations was statistically significant (Table 3).

**Table 3:** Summary of the associations between *self-reported* measures of built characteristics and physical activity in adults with low SES

Study	Built characteristics	Type of PA	Measurements of PA	Estimates (95% CI)
Chudyk et al. (2017)	*NEWS-A* Aesthetics Crime Traffic	*Physical Activity*	*Physical Activity*	
		Total activity counts (TAC)	TAC number/day	
			Aesthetics	*β* = 1.08 (0.94–1.23)
			Crime	*β* = 1.07 (0.95–1.22)
		Steps	Number of steps/day	
			Aesthetics	*β* = 233 (−415–881)
		Light PA	Light PA minutes/day	
			Aesthetics	*β* = 6.26 (−13.03–25.55)
			Traffic	*β* = 11.33 (−10.79–33.45)
		Moderate to Vigorous PA	MVPA minutes/day	
			Aesthetics	*β* = 1.18 (0.87–1.60)
		*Walking for transportation (TW)*	*Walking for transportation*	
		Participation	Participation (Yes/No)	
			Aesthetics	OR = 1.15 (0.50–2.61)
		Duration	TW hours/week	
			Crime	*β* = −0.76 (−1.62–0.09)
Perez et al. (2011)	*NEWS-A* Mean score	*Sedentary Behaviour*	Participation in not sufficient PA (Yes/No)	
		No vehicle access & non-native English		OR = 0.91 (0.50–1.60)
		No vehicle access and native English		OR = 0.38 (0.16–0.90)*
		Vehicle access and non-native English		OR = 0.65 (0.38–1.10)
		Vehicle access and native English speaker		OR = 1.00 (0.53–2.00)
		*Total physical activity*	MET-hours/week	
		No vehicle access & non-native English		*β* = 0.99 (0.75–1.31)
		No vehicle access and native English		*β* = 0.78 (0.58–1.05)
		Vehicle access and non-native English		*β* = 1.24 (0.94–1.65)
		Vehicle access and native English speaker		*β* = 1.08 (0.80–1.47)

*p < 0.05

Perez et al.’s (2011) study on women living in a disadvantaged neighbourhood found a significant negative association between self-reported walkability and not meeting physical activity guidelines among a subgroup of native English speakers without access to a vehicle. The same study reported null associations between self-reported walkability and participation in or duration of physical activity for the three other subgroups (Perez et al. 2011). Similarly, Chudyk et al. (2017) found no significant associations between self-reported neighbourhood aesthetics and safety with various physical activity outcomes in older adults with low incomes.

### Study quality assessment

Six studies in this review were of good quality, while one study was judged to be “fair” in quality due to unclear reporting by the authors of how exposure measures were defined and whether adjustments were made for potential confounders (Perez et al. 2011). Most studies (*n* = 6) reported the reliability and validity of their instruments for measuring the built environment. Six studies clearly described and justified their statistical analyses. Six studies adjusted for key potential confounders in the analysis (i.e., controlling for at least one socio-demographic and one behavioural variable). Most studies reported low participation rates, with only two studies reporting participation rates above 50%. Five studies did not report sample size calculations or any concerns about sample size as a limitation. All seven studies addressed the research question with an appropriate study design (Table 4).

**Table 4:** Study quality assessment

Author (year)	Was the research question or objective in this paper clearly stated?	Was the study population clearly specified and defined	Was the participation rate of eligible persons at least 50%?	Was a sample size justification, power description, or variance and effect estimates provided?	Did the study examine different levels of the exposure as related to the outcome?	Were the exposure measures clearly defined, valid, reliable, and implemented consistently across all study participants?	Were the outcome measures clearly defined, valid, reliable, and implemented consistently across all study participants?	Were key potential confounding variables adjusted for?	Overall study quality assessment
Chudyk (2015)	Yes	Yes	No	No	Yes	Yes	Yes	Yes	Good
Chudyk (2017)	Yes	Yes	No	No	Yes	Yes	Yes	Yes	Good
McCormack (2014)	Yes	Yes	No	Yes	Yes	Yes	Yes	Yes	Good
McMorris (2015)	Yes	Yes	Yes	No	Yes	Yes	NR	Yes	Good
Perez (2011)	Yes	Yes	No	No	Yes	NR	Yes	CD	Fair
Steinmetz-Wood (2015)	Yes	Yes	NR	No	Yes	Yes	Yes	Yes	Good
Winters (2015)	Yes	Yes	Yes	Yes	Yes	Yes	NR	Yes	Good

*CD*, cannot be determined

*NR*, not reported

## Discussion

To our knowledge, this systematic review is the first to synthesize evidence on the associations between the built environment and physical activity among adults with low SES residing in Canada. Although our review found only seven studies, the findings indicate that certain built characteristics are supportive of physical activity within this population. Consistent with previous reviews on the built environment and physical activity among general adult populations (Hajna et al. 2015; Ding et al. 2018; Farkas et al. 2019), we found objectively measured street connectivity, greenness, density of destinations, and overall walkability were positively associated with physical activity among adults with low SES. In contrast to prior research (Orstad et al. 2017), associations in our review were more consistent when the built environment was objectively measured rather than self-reported.

A previous review examining socio-economically disadvantaged populations in the USA (Lovasi et al. 2009) found that self-reported proximity to trails, places to exercise, and enjoyable scenery were important built characteristics associated with physical activity. We found that, among socio-economically disadvantaged adults in Canada, street connectivity, greenness, density of destinations, and overall walkability were associated with physical activity. This difference in findings highlights the need for reviews of geographically specific evidence and the need for future Canadian studies to investigate a wider variety of built characteristics in relation to physical activity among adults with low SES.

Despite our focus on adults with low SES, our findings are consistent with a review of objective measures of the built environment and walking among the general Canadian adult population (Farkas et al. 2019). Farkas et al. (2019) found consistent associations between land-use mix, proximity to destinations, and overall walkability with walking. Among the studies in our review that examined walking as an outcome, we found positive associations between objectively measured walkability and walking among adults with low SES. Differences in findings may be partially explained by the fact that Farkas et al.’s (2019) review included more studies and focused on the general adult population in Canada.

The studies included in our review were primarily undertaken in Western Canadian provinces (i.e., British Columbia and Alberta), with fewer undertaken in Eastern Canadian provinces (i.e., Ontario and Quebec), and only one study included participants from across Canada. All the studies in this review were undertaken in urban settings only. As a result, these findings may be less generalizable to Eastern provinces, the territories, and rural settings.

In our review, we found that associations with physical activity were more consistent when the built environment was objectively measured compared with self-reported. This pattern of associations is contrary to prior research. A recent systematic review found that self-reported environment variables were associated with physical activity at higher rates than objectively measured environment variables (Orstad et al. 2017). However, there were only 2 studies included in our review that collected self-reported built characteristics and they had smaller samples (≤ 161 participants; Perez et al. 2011; Chudyk et al. 2017) relative to the studies that included objective measures of the built environment (305 to 37,241 participants; Winters et al. 2015; Steinmetz-Wood and Kestens 2015; McMorris et al. 2015; McCormack et al. 2014). Thus, the pattern of associations could have been partly because most studies in our review used objective measures or because the studies with self-reported built environment measurements may have been unpowered to detect statistically significant associations. Alternatively, self-reports and objective measures of the environment may be distinct constructs which are differentially associated with physical activity. Low agreement between self-reports and objective measures of the same built characteristics is often found, providing support for the two being distinct constructs (Orstad et al. 2017; Leslie et al. 2010). If the two types of measures represent distinct constructs, then different interventions might be designed: for example, altering people’s perceptions of the built environment instead of altering the actual environment. Future studies should consider including both self-report and objective measures of the built environment to improve understanding of their relative contributions to physical activity among adults with low SES.

None of the studies included in this review controlled for residential self-selection, which may have biased the individual study results and the overall conclusions of the review. For example, if individuals who are inclined to walk in their neighbourhood specifically seek out housing in neighbourhoods that cater to that desire, then the magnitude of any association between the built environment and physical activity is likely to be overestimated (McCormack and Shiell 2011; Cao et al. 2009). This bias may be less of an issue for research on people with low SES because their neighbourhood choices are more likely constrained by financial reasons. Thus, it is less likely that adults with low SES are choosing neighbourhoods primarily for their physical activity supportiveness. Residential relocation studies (that monitor physical activity levels before and after neighbourhood relocation) and natural experiments (pre- and post-intervention studies following the same people in the neighbourhood) can help to address this limitation (Gebel et al. 2015). Alternatively, studies targeting individuals with low SES could recruit participants living in affordable housing to reduce the risk of self-selection bias because individuals moving into affordable housing often have little choice about what type of neighbourhood the housing they are offered is located within.

Notably, only three studies were specifically designed to examine the relation between built environment and physical activity among people with low SES (Chudyk et al. 2017; Chudyk et al. 2015; Perez et al. 2011). The other four studies recruited a heterogeneous sample and then stratified or tested for modification by SES (McMorris et al. 2015; Steinmetz-Wood and Kestens 2015; McCormack et al. 2014; Winters et al. 2015). The studies with heterogeneous samples often had few participants with low SES, which meant the researcher-aggregated low-SES groups may not be representative of a low-SES population. For example, one study combined the two lowest income adequacy groups because there were too few participants in the lowest group (McMorris et al. 2015), and another used the median neighbourhood deprivation score to split the participants into high- and low-SES groups (Steinmetz-Wood and Kestens 2015). Future research should consider using policy-relevant cut-points that do not rely on the sample distribution. For example, Winters et al. (2015) divided their sample based on the median household income for Canadian older adults. Several authors have noted the difficulty of recruiting adults with low SES. Perez et al. (2011) undertook multiple attempts (by mail and in-person) to recruit their participants. Chudyk et al. (2015) mailed eligible adults information about the study and followed up with telephone calls to non-respondents. More intensive recruitment approaches might be needed to recruit adults with low SES.

Overall, the studies included in this review were of good quality. Of concern were the small sample sizes, which may partially explain the large number of null results. The generalizability of the results presented in this review is limited by the low participation rates and the lack of information reported in the studies related to the low-SES subsample participant characteristics. Similar to previous reviews (Kärmeniemi et al. 2018; Ding et al. 2018), there was heterogeneity in measures of both exposures and outcomes across studies, making direct comparison between studies challenging.

### Strengths and limitations

A strength of this systematic review is adherence to the PRISMA statement, including a study quality assessment (Moher et al. 2009), which has rarely been done previously for reviews on this topic (Ding and Gebel 2012). Another strength was our comprehensive search strategy, reducing the possibility of missing relevant studies. Our focus on Canadian studies provides useful evidence that can inform local urban planning and public health-related policy and practice.

A limitation of our review is the inclusion of only peer-reviewed literature, which may have introduced publication bias given that null or unexpected results are less likely to be published in peer-reviewed publications. In addition, the small number of studies included in the review, combined with many different operational definitions of the built environment and physical activity, did not allow us to categorize results by specific built characteristics or physical activity types. We acknowledge that specific types of built characteristics (e.g., mixed land use) might be important for supporting specific types of physical activity (e.g., walking for transport; Giles-Corti et al. 2005). Studies with increased specificity of the built environment and physical activity types among low-SES populations in the Canadian context are needed.

The small number of studies included in the review also limited our ability to specifically evaluate built environment and physical activity associations in relation to the different measures of SES used (e.g., individual level vs neighbourhood level, or income vs education). We acknowledge that neighbourhood-level SES is often a poor proxy for individual-level SES (Hanley and Morgan 2008), particularly when there is heterogeneity of SES within neighbourhoods (Diez Roux 2004) and that different indicators (e.g., education or income) may contribute to physical activity in different ways (Gidlow et al. 2006). Future reviews that include a sufficient number of studies should consider exploring the role of different SES indicators in explaining built environment–physical activity relationships.

Another important consideration is the extent to which access to physical activity supportive built environments differ by SES. Similar to research in Canada on access to healthy food environments by SES (e.g., Minaker et al. 2016), there are mixed findings in Canada related to access to physical activity supportive environments. For example, there is some evidence that adults with lower incomes are more likely (than those with high incomes) to live in neighbourhoods with higher walkability levels (McCormack et al. 2014), but there is also evidence that adults in Canada with lower incomes are more likely to live in urban areas with less green space (Villeneuve et al. 2012; McMorris et al. 2015).

## Conclusion

This review found a paucity of quantitative research examining associations between the built environment and physical activity among adults with low SES in Canada. However, the seven available studies for review suggest that objectively measured street connectivity, greenness, density of destinations, and overall walkability (self-reported or objectively measured) are associated with physical activity among adults with low SES. Future Canadian studies should specifically recruit participants with low SES, adjust for residential self-selection, examine longitudinal or temporal associations, and take neighbourhood exposure time into account.

## Appendix 1. Detailed search strategy example (Medline)

Database(s): **Ovid MEDLINE(R) and Epub Ahead of Print, In-Process & Other Non-Indexed Citations and Daily** 1946 to October 18, 2018

Search Strategy:

**Table:**

#	Searches	Results
1	Canada/	84,637
2	New Brunswick/	686
3	Prince Edward Island/	284
4	Alberta/	7341
5	British Columbia/	9471
6	Newfoundland/	1290
7	Labrador/	1290
8	Manitoba/	3104
9	Ontario/	25,022
10	Quebec/	13,084
11	Nova Scotia/	2324
12	Saskatchewan/	2408
13	Yukon/	182
14	Northwest Territories/	355
15	Nunavut/	291
16	1 or 2 or 3 or 4 or 5 or 6 or 7 or 8 or 9 or 10 or 11 or 12 or 13 or 14 or 15	146,284
17	(Canad* or New Brunswick or Prince Edward Island or Alberta or British Columbia or Newfoundland or Labrador or Manitoba or Ontario or Quebec or Nova Scotia or Saskatchewan or Yukon or Northwest Territories or Nunavut).tw,kf.	148,450
18	16 or 17	214,530
19	Environment Design/	5682
20	(Walkab* or pedestrian* or (land adj2 mix) or traffic or road).tw,kf.	67,499
21	(density adj5 (residential or employment or intersection*)).tw,kf.	571
22	(proximity adj5 (amenit* or service* or transit or park*)).tw,kf.	282
23	(connect* adj5 (road and roads or street*)).tw,kf.	303
24	(destination* or environmental modification*).tw,kf.	10,983
25	(footpath* or sidewalk* or walking track*).tw,kf.	1056
26	cycl*lane*.tw,kf.	29
27	(bike adj2 path*).tw,kf.	37
28	(cycl* adj2 (path* or track*)).tw,kf.	7511
29	((green adj2 space*) or greenness or public space* or public open space* or park* or (park adj2 land) or playground or (play* adj2 area*)).tw,kf.	132,066
30	(urban sprawl or car dependent).tw,kf.	340
31	(physical activity environment* or active environment* or healthy place* or urban environment design or pedestrian environment or physical environment or urban form or urban design or infastructure or neighbourhood* or neighbourhood* or aesthetic* or street lighting* or surrounding* or communit* or obesogenic or Walk score).tw,kf.	707,227
32	19 or 20 or 21 or 22 or 23 or 24 or 25 or 26 or 27 or 28 or 29 or 30 or 31	913,281
33	(Walk* or div* or walking trip*).tw,kf.	474,396
34	(commut* or (active travel or active living or active transport)).tw,kf.	11,673
35	(physically active or physically inactive or physical inactivity or physical activity).tw,kf.	99,488
36	(physical adj3 activit*).tw,kf.	98,608
37	aerobic.tw,kf.	75,663
38	(physical exertion or physical fitness or acute exercis*).tw,kf.	13,537
39	(training or cycle or bicycle or cycling or sports or exercis* or steps).tw,kf.	1,226,179
40	non motorized mode.tw,kf	1
41	(travel behaviour or pedestrian behaviour or pedestrian travel or pedestrian transportation).tw,kf.	276
42	(jogging or jog or jogger* or running or run or runner*).tw,kf.	129,769
43	exercise/ or physical conditioning, human/ or running/ or walking/	136,633
44	33 or 34 or 35 or 36 or 37 or 38 or 39 or 40 or 41 or 42 or 43	1,878,668
45	18 and 32 and 44	4757

## Appendix 2. Modified National Heart, Lung and Blood Institute Quality Assessment Tool for Observational Cohort and Cross-Sectional Studies

Response options: Yes, No, (CD, NR, NA)*

1. Was the research question or objective in this paper clearly stated?
2. Was the study population clearly specified and defined?
3. Was the participation rate of eligible persons at least 50%?
4. Was a sample size justification, power description, or variance and effect estimates provided?
5. For exposures that can vary in amount or level, did the study examine different levels of the exposure as related to the outcome (e.g., categories of exposure, or exposure measured as continuous variable)?
6. Were the exposure measures (independent variables) clearly defined, valid, reliable, and implemented consistently across all study participants?
7. Were the outcome measures (dependent variables) clearly defined, valid, reliable, and implemented consistently across all study participants?
8. Were key potential confounding variables measured and adjusted statistically for their impact on the relationship between exposure(s) and outcome(s)?

*CD, cannot determine; NA, not applicable; NR, not reported
